# New-Onset Atrial Fibrillation in the Critically Ill COVID-19 Patients Hospitalized in the Intensive Care Unit

**DOI:** 10.3390/jcm12226989

**Published:** 2023-11-08

**Authors:** George E. Zakynthinos, Vasiliki Tsolaki, Evangelos Oikonomou, Manolis Vavouranakis, Gerasimos Siasos, Epaminondas Zakynthinos

**Affiliations:** 1 3rd Department of Cardiology, “Sotiria” Chest Diseases Hospital, Medical School, National and Kapodistrian University of Athens, 11527 Athens, Greece; gzakynthinos2@gmail.com (G.E.Z.); boikono@gmail.com (E.O.); vavouranakis@gmail.com (M.V.); ger_sias@hotmail.com (G.S.); 2 Critical Care Department, University Hospital of Larissa, Faculty of Medicine, University of Thessaly, 41110 Larissa, Greece; vasotsolaki@yahoo.com; 3 Cardiovascular Division, Brigham and Women’s Hospital, Harvard Medical School, Boston, MA 02115, USA

**Keywords:** new-onset atrial fibrillation, COVID-19, ICU, critical illness, trigger factors

## Abstract

New-onset atrial fibrillation (NOAF) is the most frequently encountered cardiac arrhythmia observed in patients with COVID-19 infection, particularly in Intensive Care Unit (ICU) patients. The purpose of the present review is to delve into the occurrence of NOAF in COVID-19 and thoroughly review recent, pertinent data. However, the causality behind this connection has yet to be thoroughly explored. The proposed mechanisms that could contribute to the development of AF in these patients include myocardial damage resulting from direct virus-induced cardiac injury, potentially leading to perimyocarditis; a cytokine crisis and heightened inflammatory response; hypoxemia due to acute respiratory distress; disturbances in acid-base and electrolyte levels; as well as the frequent use of adrenergic drugs in critically ill patients. Additionally, secondary bacterial sepsis and septic shock have been suggested as primary causes of NOAF in ICU patients. This notion gains strength from the observation of a similar prevalence of NOAF in septic non-COVID ICU patients with ARDS. It is plausible that both myocardial involvement from SARS-CoV-2 and secondary sepsis play pivotal roles in the onset of arrhythmia in ICU patients. Nonetheless, there exists a significant variation in the prevalence of NOAF among studies focused on severe COVID-19 cases with ARDS. This discrepancy could be attributed to the inclusion of mixed populations with varying degrees of illness severity, encompassing not only patients in general wards but also those admitted to the ICU, whether intubated or not. Furthermore, the occurrence of NOAF is linked to increased morbidity and mortality. However, it remains to be determined whether NOAF independently influences outcomes in critically ill COVID-19 ICU patients or if it merely reflects the disease’s severity. Lastly, the management of NOAF in these patients has not been extensively studied. Nevertheless, the current guidelines for NOAF in non-COVID ICU patients appear to be effective, while accounting for the specific drugs used in COVID-19 treatment that may prolong the QT interval (although drugs like lopinavir/ritonavir, hydrochlorothiazide, and azithromycin have been discontinued) or induce bradycardia (e.g., remdesivir).

## 1. Introduction

The primary manifestation of Coronavirus Disease 2019 (COVID-19) is pneumonia, which can lead to Acute Respiratory Distress Syndrome (ARDS) in 6% of cases [1,2,3]. Moreover, cardiovascular complications are frequently observed in COVID-19 patients, although, cardiac involvement is primarily indicated by mildly elevated troponin levels [4,5,6,7,8]. Cardiac arrhythmias, particularly new-onset atrial fibrillation (NOAF), have been documented in COVID-19 patients, but the incidence varies widely, ranging from 3% to 10% in patients hospitalized in general wards, and from 16% to 44% in Intensive Care Unit (ICU) patients [9,10,11,12,13,14,15,16]. Hence, NOAF is common in ICU COVID-19 patients and tends to manifest later in the disease course, typically after respiratory deterioration leading to ICU admission [1,14,15]. Notably, NOAF has been identified in severe COVID-19 cases, often after the patients are already admitted to the ICU [4].

COVID-19 per se could be the cause, but in ICU patients, multiple triggers may be implicated in AF occurrence. Pre-existing AF is typically found among the elderly population with multiple comorbidities, having numerous risk factors for intensive care admission. Nonetheless, NOAF can emerge during critical illness in patients with no prior history of AF, often lacking significant cardiac disease or structural changes [17]. Therefore, it is suggested that the pathophysiology and causes of NOAF during critical illness differ from AF in non-critically ill patients, primarily due to the presence of reversible factors like hypoxemia, inflammation, electrolyte imbalances, fluid overload, excessive adrenergic stimulation, and the use of vasopressors [17,18].

NOAF is believed to be the most prevalent arrhythmia in non-COVID-19 ICU patients, occurring in 19–35% of cases, with sepsis being a key triggering factor [17,19,20]. Walkey et al. reported a heightened incidence (35%) of NOAF among septic patients, which further rises with disease severity [19]. Given that all these factors exist in non-COVID-19 ICU patients, they are also relevant to patients with COVID-19. We recently proposed that both myocardial involvement due to SARS-CoV-2 infection and sepsis participate in the onset of NOAF in intubated ARDS patients, particularly later in the disease course [21].

Furthermore, NOAF appears to be linked to adverse outcomes, including mortality, increased ICU stays, and longer hospital stays [22,23,24]. However, it remains uncertain whether these adverse outcomes are related to NOAF occurrence per se or are depicted in the increased disease severity.

Nevertheless, evidence regarding the optimal management strategy for NOAF in the ICU setting, whether in COVID-19 or non-COVID-19 patients, is limited [17,18]. However, critical care guidelines for AF should be followed in cases of NOAF in COVID-19 patients, considering the co-administration of COVID-19 drugs. Direct cardioversion should be attempted in cases of hemodynamic instability, and amiodarone infusion appears to be one of the preferred treatment options for rate and rhythm control in case of heart failure, otherwise, b-blockers or calcium channel blockers may be used for rate control [25].

In this literature review, our objective is to summarize the most recent evidence regarding the incidence of NOAF in critically ill COVID-19 ICU patients and analyze the current state of knowledge regarding the underlying mechanisms leading to its occurrence. Specifically, we aim to investigate existing data and diagnostic evidence that could support the presence of cardiac damage from the virus itself, ARDS, mechanical ventilation, and other risk factors common in the ICU, and their association with NOAF. Additionally, we intend to present the prognostic significance of NOAF on mortality and explore the current management of NOAF in the ICU.

## 2. Incidence of New-Onset Atrial Fibrillation in Patients with Severe COVID-19

Cardiovascular diseases have emerged as prominent contributors to clinical deterioration and unfavorable outcomes in COVID-19 patients [26]. The correlation between COVID-19 and cardiac arrhythmias has also been highlighted (Table 1) [11]. In a cohort study involving 137 patients admitted to tertiary hospitals in China, 7.3% of them initially presented with heart palpitations [27]. Atrial fibrillation (AF) is the most prevalent form of atrial arrhythmia in COVID-19 patients, with the prevalence of NOAF ranging from 3% to 10% in non-ICU patients [10,11].

In an initial study conducted by Wang et al., it was observed that arrhythmias were more frequent among ICU patients with COVID-19 (44.4%) compared to non-ICU patients (6.9%); however, the specific nature of these arrhythmias was not detailed [5]. In a retrospective multicenter study by Kantasamy et al., which encompassed 109 consecutive patients admitted to the ICU with confirmed COVID-19 pneumonia and a definitive outcome (death or discharge), 14.6% developed NOAF during their ICU stay. This risk was more pronounced in older patients with underlying chronic heart failure and chronic kidney disease. It is worth noting that three-fourths of the population had two or more comorbidities [28].

In a study conducted by Colon et al., which involved 115 COVID-19 patients admitted to the hospital, including 69 in the medical ICU and 46 in a general medicine ward, a tachyarrhythmia, primarily NOAF, that was not present upon admission was identified in 19 patients (16.5%). All these cases were among ICU patients (27.5% of ICU patients), while no such arrhythmias were reported among patients admitted to general wards [13]. A multicenter cohort study covering critically ill adult patients admitted to ICUs in five hospitals in Saudi Arabia found that 10.7% developed NOAF during their ICU stay [29].

In our recent prospective study [21], focusing solely on intubated patients with COVID-19-induced ARDS, which represents the most severely ill ICU population, the incidence of NOAF was 24%. This aligns with the higher incidences reported in ICU COVID-19 patients [9,11,12,15,16]. Our study demonstrated that factors like hypertension history, time since COVID-19 onset, ferritin levels, and corticosteroid treatment, did not significantly influence NOAF occurrence. Additionally, demographic details, Charlson comorbidity index, ARDS severity, respiratory mechanics, mechanical ventilation modes, and electrolyte levels did not exhibit notable differences between the groups. Importantly, our study stands out as the only one, to our knowledge, focusing exclusively on intubated severe ARDS patients, presenting detailed information on mechanical ventilation modes, which could impact cardiac function and, consequently, arrhythmia occurrence.

In another retrospective study encompassing ICU-admitted COVID-19 patients, the incidence of NOAF was 14.9%, with the median age of the NOAF group being 79.0 years. The risk of NOAF correlated with older age and the presence of comorbidities [30]. Similarly, among COVID-19 patients admitted to two hospitals in New York City, atrial arrhythmias (without specifying the type) were reported in 17.7% of the patients who received mechanical ventilation, in contrast to 1.9% of patients without mechanical ventilation [14].

In their meta-analysis of 31 studies exploring NOAF in hospitalized COVID-19 patients, Romiti et al. found that NOAF was present in 8% of cases, suggesting that the actual prevalence of atrial fibrillation could be as high as 27%, considering the substantial heterogeneity among the studies. AF in COVID-19 patients was more likely to occur in older individuals with conditions such as hypertension, diabetes, concurrent coronary artery disease, congestive heart failure, and critical clinical status [31]. It may reflect a direct viral invasion or a result of RV dysfunction, yet NOAF is frequently encountered in nonCOVID-19 ARDS ICU patients.

NOAF is a common complication of critical illness, emerging as the predominant cardiac arrhythmia in non-COVID-19 ICUs [39,40], and significantly impacting management and prognosis [41,42].

In an early study, ICU physicians observed NOAF in almost one out of three critically ill patients [43]. This percentage remains consistent in subsequent studies as well. The incidence of NOAF in mixed ICUs, encompassing both surgical and medical cases, varies widely in the literature, ranging between 19% and 35% [17,18,19,20,39,40,41,42].

As suggested by the aforementioned findings, the prevalence of NOAF in COVID-19 patients does not significantly differ from that observed in non-COVID-19 ICU patients with ARDS. However, there exists a substantial variation in the reported prevalence across different studies. In patients with community acquired pneumonia, NOAF occurrence has been reported in 7–12%; yet most of the patients were being hospitalized in the general ward. Patients with severe CAP presented a higher NOAF incidence of 23% [44,45,46]. Additionally, one study claimed that the incidence of NOAF was comparably lower in COVID-19 patients compared to those treated in the ICU for severe sepsis [29]. It is important to note that the age of the COVID-19 patients in this study was lower than that of patients admitted to general ICUs, and it is well established that age, among other factors, influences the occurrence of arrhythmias [17].

Lastly, only a limited number of studies have reported on the occurrence of NOAF in ICU patients with COVID-19-related ARDS (Table 1), and most of these studies are retrospective in nature. Moreover, these studies often include a mixed population of patients from both wards and ICUs, failing to distinguish between intubated and non-intubated ICU patients. Furthermore, the initial studies did not provide detailed insights into the specific types of arrhythmias observed (Table 1).

## 3. Pathophysiological Mechanisms Assumed to Be Involved in the Appearance of NOAF

In the available literature, the specific types and underlying mechanisms of reported arrhythmias related to critically ill patients with COVID-19 have not yet been thoroughly elucidated.

**Figure 1:**
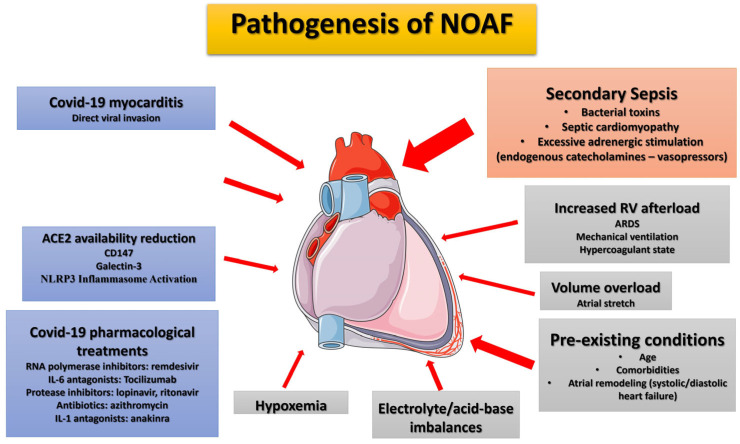
Proposed pathophysiological mechanisms and risk factors for new-onset atrial fibrillation in ICU COVID-19 ARDS patients. During SARS-CoV-2 infection, chronic factors (age, pre-existing comorbidities), COVID-19-specific factors (inflammation, cytokine storm, direct viral invasion, treatments received)) and ICU risk factors (secondary sepsis, associated with adrenergic-endogenous/exogenous-overstimulation, right ventricular overload: ARDS/mechanical ventilation, hypoxemia, electrolyte imbalances, and acid-base disturbances) may contribute to NOAF emergence. The most significant factor in ICU patients, in the authors’ opinion, is secondary sepsis (orange box) which typically occurs relatively late in the course since SARS-CoV-2 infection. The arrow width indicates the degree of contribution of each factor in NOAF emergence. ACE2, angiotensin converting enzyme 2; ARDS, acute respiratory distress syndrome; COVID-19, Corona Virus Disease 2019; IL-1, interleucin-1; IL-6, interleukin-6; NLRP3, NOD-like receptor pyrin domain-containing 3; NOAF, new-onset atrial fibrillation; RNA, rivonucleic acid; RV, right ventricle.

There are numerous proposed pathogenetic mechanisms, including direct viral myocardial injury (myocarditis), imbalances in myocardial oxygen supply and demand, hypoxemia due to acute respiratory distress, an increased inflammatory response, elevated right ventricular afterload, diffuse endothelialitis, procoagulant activity, acid-base imbalances, and electrolyte abnormalities (Figure 1) [4,47,48].

Myocarditis could reasonably serve as a primary explanation for new-onset arrhythmias in COVID-19 patients who undergo cardiac injury, leading to the development of arrhythmias. A study revealed that the prevalence of heart failure was 23% among patients with COVID-19 [49]. However, it remains unclear whether the new cardiomyopathy (potentially due to myocarditis) or the exacerbation of underlying myocardial dysfunction could account for the high prevalence of heart failure reported in a few studies within this population [50,51].

Undoubtedly, the COVID-19 virus invades the myocardium. An initial post-mortem pathological study discovered myocardial tissue positive for SARS-CoV-2 through reverse transcription polymerase chain reaction (RTPCR) and electron microscopy in one out of four patients examined [52]. Nevertheless, cardiac involvement is primarily based on mildly increased troponin levels in most studies [5,6,7,8]. More detailed echocardiographic examinations are relatively scarce [53,54], with conflicting results concerning the correlation between increased troponin levels and cardiac function impairment [55,56].

Even fewer studies present echocardiographic data in critically ill COVID-19 patients, especially without differentiating between mechanically ventilated (MV) and non-MV patients, which represent the more severe COVID-19 cases. Furthermore, when these data are presented, they often lack information regarding the presence of arrhythmias, including NOAF, and their association with heart function. However, it appears that both the right and left ventricles are significantly impaired in COVID-19 patients.

The ECHO-COVID study, a multicenter research effort across European ICUs employing conventional echocardiography, reported that among the 69% of MV patients, 34.5% exhibited right ventricular (RV) dysfunction, and 22% displayed left ventricular (LV) systolic dysfunction, based on rough visual estimation. However, 30% of these patients had a previous history of cardiomyopathy and were not excluded from the study [57].

Notably, speckle-tracking echocardiography seems to be a more reliable method for assessing myocardial damage. This approach showed severe impairment in most COVID-19 patients, even when the ejection fraction (EF) seemed normal. For instance, Janus et al. discovered a severely depressed global longitudinal strain of the left ventricle (LV-LS) at −11.8% in 31 COVID-19 patients, which is severely depressed although concerned COVID -19 patients hospitalized in the general ward, obviously without severe ARDS [58]. Similarly, Van den Heuvel et al. assessed LV function using strain imaging in 51 patients, 17 of whom required MV. Among them, 14 patients (27%) displayed impaired LV function, with 11 having a low LV-LS and a normal EF [55]. In a multicenter study by Karagodin et al., in a large multicenter study around the world, the mean LV-LS was reported at −17.9% in ICU patients. However, only a subset of these patients required mechanical ventilation (15% in the whole cohort) [59].

During the pandemic, the right ventricle has been widely recognized as one of the most frequently affected cardiovascular areas in COVID-19 [48,60,61]. This, as already reported in the ECHO-COVID study, employing only conventional echocardiography, has been noted [57]. The reported prevalence of right ventricular dysfunction ranges from 6% to almost 50%, the wide variability being explained by differences in the severity of the included population and the definitions used for ventricular dysfunction [62,63]. Current COVID-19 data using comprehensive echocardiography, especially in the most severe subset of patients, indicate a significant impairment of the right ventricular strain in more severe patients. Specifically, the RV longitudinal strain (RV-LS) has been found to be lower in patients with COVID-19-related ARDS compared to those without ARDS (−21.3% vs. −24.6%), in patients in the ICU compared to those not in the ICU (−17.5% vs. −19.8%), and in non-survivors compared to survivors (−19% vs. −14%) [64,65,66]. However, the mechanical ventilator settings and respiratory system mechanics were not reported, which prevents conclusive remarks on the effects of mechanical ventilation on the observed outcomes. Moreover, an association between right ventricular dysfunction and increased mortality has also been proposed [63,67].

The involvement of the right ventricle in COVID-19 ARDS is likely multifactorial, possibly more than the left ventricle. This is due to factors such as the disease itself, increased afterload resulting from lung impairment, pulmonary vascular microthrombosis, and the potential impact of mechanical ventilator settings. It is widely acknowledged that mechanical ventilation can notably affect both RV myocardial performance and RV afterload. When airway pressure is not restricted, it often leads to pulmonary arterial hypertension [68]. Notably, Schmitt et al. have pointed out that positive end-expiratory pressure (PEEP) obstructs RV outflow and influences RV systolic function, possibly by escalating pulmonary vascular resistance [69]. This phenomenon is likely exacerbated in COVID-19 ARDS, where there is an increased occurrence of dead space formation and relatively preserved compliance of the respiratory system [70,71]. Recent findings indicate that in COVID-19 ARDS patients with mostly preserved respiratory system mechanics, PEEP impairs RV function and has an amplified effect on RV afterload by inducing non-zone three conditions [72].

Pericardial effusion serves as a direct indicator of cardiac involvement in COVID-19 patients. It might also reflect the severity of infection, as it has been more frequently reported in ICU patients compared to non-ICU patients (23.2% vs. 16.3%) [59].

Considering the aforementioned factors and the significant impact on cardiac function during the COVID-19 era, especially in critically ill patients, one would expect an increased incidence of NOAF in such cases. Conversely, patients in the ICU who experience NOAF may demonstrate more compromised cardiac function, which could be evident through troponin levels, echocardiography, and other examinations.

Indeed, NOAF is increased in critically ill patients, especially those on mechanical ventilation, when compared to less severe patients, as previously mentioned [11,13]. However, there is no strong association between heart impairment and NOAF.

Therefore, Kanthasamy et al. [29] found that over 90% of severe COVID-19 patients in their study had elevated troponin levels above the normal range. However, they did not observe a significant association with NOAF or left ventricular systolic function (Table 1).

The meta-analysis of Romiti et al. [31], which included a large number of COVID-19 patients with an increased incidence of NOAF, did not find any difference in new heart function impairment.

Similarly, in our study involving severely ill COVID-19 ARDS ICU patients, NOAF developed late in the ICU course (18 ± 4.8 days from COVID-19 symptom onset and on day 8.5 ± 2.1 of ICU stay). Myocardial dysfunction was present on ICU admission in these patients, despite having no history of cardiac disease, as depicted by the echocardiographic findings—mainly through LV and RV longitudinal strain—of impaired left and right ventricular function, mild pericardial effusion, and mild elevation of troponin levels. However, NOAF was found to be independently associated only with secondary infections that led to severe sepsis. Notably, 84.2% of NOAF cases occurred concurrently with a septic episode, typically involving septic shock resulting from ventilator-associated pneumonia (VAP) and/or bacteremia. Our suggestion is that sepsis triggered the occurrence of NOAF, given the compromised myocardium due to COVID-19. Interestingly, this notion is reinforced by the fact that the resolution of sepsis was vital in maintaining sinus rhythm (under amiodarone infusion) [21]. Consistent with our findings, histological examinations reveal macrophage infiltration without a clear link to myocardial injury. Although troponin levels were elevated, true myocarditis was only established in 4.5% of severely ill COVID-19 patients with heart failure who underwent endomyocardial biopsies. This suggests that the virus seems to directly invade cardiac cells to initiate AF in a minority of patients [73]. In all likelihood, inflammation, sepsis, and septic shock are critical initiators of NOAF.

To our knowledge, most studies reporting NOAF in critically ill patients fail to address potential secondary conditions and the timing of NOAF onset during the course of COVID-19. Although increased inflammatory markers and the need for vasopressors were reported during AF episodes, they do not specify whether the appearance of AF coincided with a secondary infection episode [4,11,13]. Similarly, studies confirming the heightened incidence of NOAF in ICU COVID-19 patients do not specify whether the virus or other factors often present in critically ill patients are associated with its occurrence [9,10,11,12,13,14,15,16]. Just as we did, Ergun et al., who included only critically ill patients with severe illness, also found that NOAF was predominantly detected in patients who developed secondary bacterial infections during ICU follow-ups [30].

Furthermore, there have been consistent reports of troponin elevation in bacterial sepsis, indicating altered cardiomyocyte permeability or some degree of necrosis, either with or without evident cardiac dysfunction [74,75]. Additionally, sepsis-induced myocardial dysfunction is highly prevalent among ICU patients, attributed to heightened circulating levels of catecholamines and cytokines, commonly found in cases of severe sepsis and septic shock [76,77], thereby justifying an increased occurrence of arrhythmias. Nonetheless, reduced systemic vascular resistance might obscure the altered myocardial performance. Sepsis-induced vasoplegia could account for a seemingly preserved LV Ejection Fraction in such instances [21,78]. In our study, markers of inflammation, the requirement for vasopressors, and lactate levels exhibited a gradual increase leading up to the onset of NOAF, while hypoxemia notably improved in NOAF patients on the day AF manifested. However, apparent dysfunction of the RV or LV was not evident (though sepsis cardiomyopathy cannot be ruled out since both LV and RV myocardial strain were significantly impaired). Moreover, troponin levels exhibited a significant increase on the day of AF compared to admission, further supporting the connection between NOAF and secondary sepsis [21].

Consistent with the above, patients who underwent invasive mechanical ventilation were more prone to requiring vasopressor support (95.4% vs. 1.5%) and experiencing other complications, including arrhythmias [14], while cardiac arrests and arrhythmias are likely the consequence of systemic illness and not solely the direct effects of COVID-19 infection [11].

In the general ICU, NOAF occurs more frequently among septic patients receiving vasopressor agents, those with electrolyte imbalances, and those with more severe disease states [17,79,80,81]. Hypokalemia and alterations in autonomic activity balance due to vasopressors can modify ion channel behavior and cellular automaticity, increasing susceptibility to AF [82,83]. Particularly, epinephrine has chronotropic effects that can lead to increased atrial ectopic discharges, thus triggering more NOAF episodes [82]. Greater illness severity is also linked to a higher risk of NOAF development, likely due to increased release of catecholamines and progressively severe autonomic dysfunction [42,80,84]. However, phenomena such as the cytokine storm and oxidative stress, possibly contributing to atrial remodeling, are among the potential triggers for AF onset, particularly in individuals with a predisposition [40]. Elevated serum levels of inflammatory mediators, including tumor necrosis factor-alpha (TNF-α) and interleukins (IL)-1β, IL-6, IL-8, and IL-10, have been observed in non-COVID-19 patients with NOAF, showing a correlation with AF duration and severity [85]. Consequently, treatments aimed at reducing inflammatory responses and oxidative stress have shown promising results in mitigating atrial structural and electrical remodeling [86,87].

All these phenomena are also commonly reported, and indeed, more exaggerated, in COVID-19 patients [88]. COVID-19 has been characterized from the outset as an inflammatory condition [89], and targeting the dysregulated immune response has been proposed as a promising therapeutic strategy [90,91,92,93,94]. A link between AF and COVID-19 might be explained by the heightened burden of systemic inflammation, at least in the initial disease stage. Similarly, like in non-COVID-19 patients, higher levels of C-reactive protein and interleukin-6 were observed in COVID-19 patients with NOAF compared to those without AF [95]. Moreover, COVID-19 patients exhibited elevated levels of proinflammatory cytokines, such as interleukin (IL)-1, IL-6, interferon gamma (IFN-g), IFN-inducible protein-10 (IP-10), and monocyte chemoattractant protein-1 (MCP-1), possibly driving the activated T-helper-1 cell response [96]. Additionally, it was reported that patients requiring ICU admission had higher concentrations of granulocyte colony-stimulating factor (GCSF), IP10, MCP-1, macrophage inflammatory protein-1A (MIP-1A), and tumor necrosis factor-alpha (TNF-a) compared to non-ICU patients [97]. Inflammation may also trigger arrhythmias through determining a more severe clinical course of COVID-19. The inflammatory burden of a cytokine storm during COVID-19 was associated with critical illness, greater disease severity, and outcome [96,97,98].

It is worth noting that recent reports have observed an increased incidence of secondary infections in COVID-19 ARDS patients on mechanical ventilation, particularly after the seventh day in the ICU [99,100]. This may be partially attributed to the use of corticosteroids, Tocilizumab, and Anakinra in COVID-19 ARDS treatment [99,100]. Accordingly, sepsis appears to be a primary triggering factor for NOAF in non-COVID-19 ICU patients as well [17,19,20,39,40]. Therefore, the increased sepsis incidence in COVID-19 ICU patients may partly justify the increased NOAF incidence compared to non-COVID-19 ICU patients. Additionally, the increased burden of systemic inflammation may result in increased NOAF incidence in non-ICU COVID-19 patients, as increased levels of systemic inflammation are mainly encountered at the initial disease stages. 

The aforementioned data support the notion that a degree of myocardial injury is present in severe COVID-19 patients admitted to the ICU. Taken collectively, these findings may be interpreted as outcomes of a complex interplay between systemic inflammation—mainly secondary sepsis when patients are already in the ICU, which typically occurs relatively late in the course of the initial COVID-19 disease—clinical status, and the onset of AF.

In an attempt to outline the pathophysiology of COVID-19-related NOAF, several other presumed mechanisms have been proposed at the cellular level, which, however, may play a role in the early presentation of NOAF, before the progression to severe ARDS, mechanical ventilation initiation, and admission to ICU (Figure 1) [87]. They include:

(a) Reduced availability of angiotensin-converting enzyme (ACE) 2: Atrial ACE2 catabolizes transforming growth factor-β1 (TGF-β1), the principal pro-fibrotic cytokine [101]. This may underlie atrial arrhythmogenesis and potentially increase susceptibility to atrial fibrillation (AF) in COVID-19 patients [102].
(b) CD147 as an adjunctive player: CD147 facilitates SARS-CoV-2 invasion into host cells, including cardiomyocytes, by interacting with the viral spike protein [103,104]. In cardiomyocytes, CD147 strongly induces IL-18, activating matrix metalloproteinases (MMPs), and elevated circulating IL-18 levels are positively correlated with AF development [105]. Moreover, higher plasma levels of MMP-9 found in AF patients suggest that MMP-9 can serve as a marker of atrial remodeling [106]. Increased circulating MMP-9 has also been observed in COVID-19 patients [107]. CD147 also stimulates oxidative stress in cardiomyocytes and promotes negative ionotropic effects [108].
(c) Role of Galectin-3: Galectin-3 plays a role in the progression of atrial fibrosis and is involved in extracellular matrix formation. Elevated galectin-3 levels correlate with advanced AF and worse outcomes [109]. Notably, galectin-3 levels are increased in the serum of COVID-19 patients and correlate with COVID-19 severity [110,111].
(d) NLRP3 Inflammasome Activation: SARS-CoV-2, by binding to ACE2, purinergic receptors, and components of the complement-mediated pathway, stimulates the formation of the NOD-like receptor pyrin domain-containing 3 (NLRP3) inflammasome [112]. A causal link exists between NLRP3 inflammasome activation in atrial cardiomyocytes and the development of AF [85,113,114].
(e) Systemic Immune Cell Over-Activation: SARS-CoV-2 infection is characterized by systemic immune cell over-activation, leading to an imbalance between T-helper-1 and Th2 cells, elevated levels of various cytokines, including IL-1β, IL-2, IL-6, IL-7, interferons, TNF-α, monocyte chemoattractant protein-1, and macrophage inflammatory protein-1A [115,116,117]. At the cardiac level, pro-inflammatory cytokines, particularly IL-6, stimulate vascular smooth muscle proliferation, endothelial cell and platelet activation, and lead to apoptosis or necrosis of myocardial cells, which could mediate intra-atrial repolarization [118].
(f) Sympathetic Nervous System Activation: In viral infections like COVID-19, activation of the sympathetic nervous system [119] leads to increased Ca^2+^ influx and cardiomyocyte overload. Ca^2+^ release, along with the subsequent generation of delayed afterdepolarizations (DADs) and action potentials, increases the probability of AF events [120].

Another potential pathophysiological mechanism involves electrophysiological changes induced by pharmacological agents commonly used in COVID-19 treatment, which carry a risk of severe and potentially fatal cardiac dysfunction, such as torsades de pointes, ventricular tachycardia, and fibrillation (Figure 1) [32]. While drugs like hydroxychloroquine, lopinavir/ritonavir, and azithromycin (known to prolong the QT interval) have been largely abandoned, remdesivir is an authorized antiviral drug for COVID-19 treatment that requires attention [33]. COVID-19 patients with oxygen saturation levels below 94% receiving intravenous remdesivir have experienced new-onset atrial fibrillation (NOAF), which is more prevalent in patients undergoing mechanical ventilation. However, the interpretation remains inconclusive due to a small sample size, short follow-up duration, and the absence of a control group [34]. Further evidence indicates that remdesivir may act as a blocker of the atrioventricular node and could be pro-arrhythmic, especially in patients with structural heart disease [35]. Therefore, patients on remdesivir therapy, especially those with pre-existing risks, must be closely monitored.

Lastly, AF is an evolving age-related disease globally, influenced significantly by co-morbidities or lifestyle conditions such as hypertension, diabetes mellitus, obesity, chronic kidney disease, and inflammatory diseases (Figure 1) [17,36,37]. These factors also play a significant role in initiating new-onset atrial fibrillation (NOAF) in both COVID-19 and non-COVID-19 ICU patients [31].

## 4. Outcome of Severely Ill COVID-19 Patients with NOAF

Despite the association of COVID-19-related NOAF with adverse outcomes and mortality, a debate persists regarding whether SARS-CoV-2 NOAF independently influences mortality, or if NOAF merely reflects the severity of the illness.

Prior research has demonstrated a link between NOAF and worse clinical outcomes in COVID-19 patients [30,38,121,122,123,124,125,126]. However, in most of these studies, NOAF has not been identified as an independent risk factor for death, particularly among ICU patients (Table 1).

An American multicenter registry involving over 30,000 COVID-19 patients found no significant connection between NOAF and mortality, though the study encompassed both ICU and non-ICU patients [38]. Thus, results from the study by Rosenblatt et al. suggest that NOAF in COVID-19 patients is likely an indicator of disease severity rather than an independent predictor of mortality [38]. Similarly, an initial study encompassing both ICU and non-ICU patients showed that incident AF, whether new-onset or recurrent, did not increase the risk of severe adverse events like ARDS or death during hospitalization [126].

In Ergun et al.’s study [30], mortality in critically ill COVID-19 patients was higher in the NOAF group (87% vs. 67%, respectively; *p* = 0.019). Nevertheless, in multivariate analysis, NOAF did not emerge as an independent risk factor for hospital mortality.

Similarly, among intubated COVID-19 ARDS patients, we observed no difference in 28-day mortality between intubated patients with NOAF and the control group [21].

Among the 14 studies reporting on all-cause mortality in COVID-19 patients, in the meta-analysis of Romiti et al., AF correlated with a fourfold higher risk of death. However, heterogeneity existed among the studies, and as the study encompassed both ICU and non-ICU patients and critical illness is linked to a higher NOAF incidence, a safe conclusion can not be easily drawn. [31]

A recent multicenter retrospective cohort study exclusively focused on COVID-19 adult patients admitted to ICUs revealed no significant differences in 30-day mortality. The MV duration, ICU length of stay (LOS), and hospital LOS were longer in patients who developed NOAF; yet, they had finally higher odds of in-hospital mortality. However, these NOAF patients were older, exhibited higher APACHE II and Multi-Organ Dysfunction Syndrome (MODS) scores on the third ICU day, experienced acute kidney injury (AKI), and more patients required mechanical ventilation and inotropes/vasopressors within 24 h of ICU admission. These findings support the notion that NOAF actually reflects critical illness severity and serves as a prognostic marker in this context [29].

Abdulrahman et al. [121] studied 492 severe COVID-19 patients, among whom 30 were diagnosed with NOAF. After adjusting for confounders in the multivariate regression model, NOAF emerged as a risk factor for worse outcomes (mechanical ventilation or death) in ICU-admitted COVID-19 patients. Similarly, in a group of 109 consecutive patients admitted to the ICU with confirmed COVID-19 pneumonia, NOAF strongly correlated with increased in-hospital mortality (OR 5.4; 95% CI 1.7–17; *p* = 0.004) [28].

Hence, the results remain inconclusive regarding whether severely ill ICU COVID-19 patients face an increased likelihood of death due to the occurrence of NOAF. However, conflicting results are also present concerning NOAF and mortality in non-COVID contexts. 

In reality, one would expect NOAF to independently impact survival in severely ill patients, whether COVID-related or not. New AF episodes have detrimental effects, including increased heart rate, irregular rhythm, and loss of atrial systole. Consequently, NOAF development can further complicate critical illness or even impede response to therapy [127]. Cardiac output may decrease due to the loss of atrial systole and tachycardia, potentially leading to acute heart failure [127,128].

Nonetheless, certain previous studies found no independent relationships between NOAF and hospital mortality in ICU patients [24,129,130]. Furthermore, in the study by Seguin et al., NOAF development correlated with critical illness severity in a surgical ICU [24]. In a multicenter prospective study by Annane et al., involving 1341 patients in general ICUs, only ventricular arrhythmias increased the risk of death, not supraventricular arrhythmias or NOAF [129].

Conversely, other studies suggested that NOAF was linked to increased hospital mortality irrespective of critical disease severity [20,131]. The FROG-ICU study encompassed 1841 critically ill patients. During their ICU stay, NOAF occurred in 12% of patients, with a 47% in-hospital mortality rate, as opposed to 23% in patients without AF. After multivariable adjustment, NOAF exhibited an increased risk of in-hospital death compared to no AF (adjusted odds ratio (OR) 1.6, *p* = 0.003) [20].

Likely, the varying percentages of NOAF occurrence in different studies (ranging from 5% to 35%), coupled with differences in shock incidence, levels of hypoxemia, and other factors, as well as variations in the timing of outcomes/mortality assessment (e.g., ICU mortality, 28-day mortality, or hospital mortality), could contribute to the disparity in the independent role of NOAF in survival.

Klein Klouwenberg et al. [42] developed a prediction tool for NOAF based on clinical factors. These factors include time since admission, age, obesity, immunocompromised state, elevation of inflammatory markers, shock, renal failure, potassium level, and fraction of inspired oxygen (FIO2). This tool has the ability to identify the risk of atrial fibrillation in sepsis patients, yielding a C-statistic of 0.81. To our knowledge, this tool has not been validated in COVID-19 ICU or non-ICU patients. It might have rendered interesting data if this tool had been used in COVID-19 ARDS patients, presenting an increased (compared to non-COVID-19 ARDS) NOAF incidence.

## 5. Management of Severely Ill COVID-19 Patients with NOAF

The management approach to new-onset atrial fibrillation (NOAF) in ICU patients depends on various factors, including underlying causes, comorbidities, hemodynamic impacts, and potential risks of treatment options. A comprehensive ICU strategy for NOAF should encompass the following: (1) assessment for potential hemodynamic effects attributable to AF; (2) correction of reversible arrhythmogenic triggers (acid-base and electrolyte abnormalities, hypoxemia if possible, overload-atrial stretch), and removal of offending agents that increase the risk for AF (i.e., beta-agonists); and (3) selection of an initial treatment strategy that maximizes potential benefit and minimizes risk when AF seems to be causing harm [17]. In cases of NOAF associated with hemodynamic compromise, synchronized direct current cardioversion should be utilized to restore sinus rhythm [25,132]. For other instances, it is reasonable to begin with a rate control strategy using beta-blockers (when not contraindicated, e.g., bronchospasm, acute heart failure), calcium channel blockers (in the absence of heart failure), and/or digoxin. In the presence of heart failure, digoxin and/or amiodarone may be considered for rate control. If NOAF has occurred within the last 48 h, the goal is to restore sinus rhythm. Considering interactions with concurrent COVID-19 pharmacotherapies, amiodarone appears to be a favorable option, especially in the presence of underlying structural heart disease or heart failure. It is essential to measure the QT interval before initiating amiodarone, particularly when co-administering QT-prolonging drugs [25]. Other antiarrhythmic drugs (AADs) are also viable options.

Class IC agents, such as flecainide and propafenone, are use-dependent INa blockers with moderate negative inotropic effects [133]. Due to their potential for proarrhythmia, they are limited to patients without coronary artery disease or structural heart issues [134]. Given their arrhythmogenic and negative inotropic effects, it is advisable not to administer them to COVID-19 patients, particularly those in critical condition or prone to myocarditis and heart failure [135]. These agents have interactions with CYP2D6 inducers and inhibitors, necessitating close electrocardiographic monitoring of the QRS complex due to QRS prolongation [133].

Beta-blockers can be employed for rate control in COVID-19 ICU patients with NOAF [134]. However, they should be avoided in patients exhibiting symptoms of acute heart failure or bronchospasm [136]. In cases of bronchospasm, calcium channel blockers like verapamil and diltiazem can serve as rate-control agents for NOAF, excluding patients with heart failure or left ventricular dysfunction due to their negative inotropic effects [134,137].

Among class III agents, amiodarone, a multichannel blocker, is highly effective and widely used in ICU patients despite its known toxicity [138,139,140,141]. Although it prolongs the QT interval, it seldom causes polymorphic ventricular tachycardia (Torsade des pointes) [133,142]. It is preferred for AF rate and rhythm control in critically ill COVID-19 patients with myocardial involvement or borderline blood pressure, as well as for NOAF prevention. Regular monitoring for liver, lung, and thyroid toxicity is recommended, especially during prolonged therapy [143]. However, due to its pulmonary toxicity, there might be concerns regarding amiodarone use in COVID-19 patients with severe pneumonia requiring prolonged mechanical ventilation [143].

The use of other class III agents, such as dronedarone, which shares similar pharmacokinetics with amiodarone [144], or dofetilide/ibutilide (IKr blockers) [145], is generally restricted. Nevertheless, ibutilide might be considered for converting acute symptomatic AF. Sotalol, a dose-dependent IKr blocker with a moderate β-blockade effect [133], is excreted through the kidneys and should thus be used with utmost caution in the ICU, where potentially nephrotoxic agents are administered; in fact, it should be considered a last resort.

For the atrial-selective class III agent Vernakalant, caution is advised in COVID-19 patients prone to hemodynamic compromise due to its known bradycardic and hypotensive effects [146], especially when using remdesivir, known to induce bradycardia, for COVID-19 treatment [147].

Digoxin can be employed in heart failure patients to control heart rate, as it lacks a negative inotropic effect. It can also be considered for patients with borderline blood pressure who cannot tolerate calcium channel blockers or β-blockers [77]. Regular monitoring of digoxin levels is recommended [25].

In COVID-19 patients with NOAF, a study from Columbia University reported that amiodarone was used in 29% of patients referred for electrophysiology consultation, and anticoagulants were employed in 83% [148]. In our study involving intubated COVID-19 ARDS patients [21], sinus rhythm was restored in 84% of the patients, with 68.4% achieving it within the first 24 h and the rest within 48 h. In all patients, amiodarone was used for cardioversion and was continued until ICU discharge or death. Only one patient out of nineteen, who experienced severe hemodynamic instability, required electrical cardioversion an hour after an unsuccessful amiodarone infusion. Interestingly, in patients where sinus rhythm was not restored, sepsis was not resolving. Furthermore, NOAF recurred in three patients, coinciding with new septic episodes, and sinus rhythm was regained upon sepsis resolution. 

Finally, NOAF is typically managed with rhythm control, preferably, or rate control, along with anticoagulation for patients who meet criteria and do not have contraindications due to bleeding risk [149].

## 6. Conclusions

New-onset atrial fibrillation is a prevalent complication in critically ill COVID-19 patients. From a clinical standpoint, data suggest that patients with severe SARS-CoV-2 infections requiring ICU care often experience myocardial injury. Elevated troponin levels and abnormal echocardiography findings, particularly in terms of longitudinal right ventricular and left ventricular strain, are observed in these patients. This phenomenon can be attributed not only to virus invasion but also to factors like sepsis, mechanical ventilation, and adrenergic overstimulation. Nonetheless, troponin levels and echocardiographic results show no significant difference between critically ill COVID-19 ARDS patients with or without the emergence of NOAF, particularly in intubated cases. Therefore, the development of NOAF in these patients likely stems from a combination of various mechanisms, including ARDS-induced hypoxemia, adrenergic stimulation, virus-related cardiac injury, electrolyte imbalances, and inherent AF susceptibility due to factors like age and pre-existing cardiovascular conditions.

We propose that secondary sepsis, accompanied by adrenergic overstimulation resulting from elevated endogenous catecholamine levels and exogenous catecholamine administration (common in septic shock), acts as the second “trigger” to induce AF in a myocardium already affected by SARS-CoV-2. This is especially evident when NOAF occurs later in the course from symptom onset. It is important to consider sepsis as a potential cause when NOAF emerges in severely ill COVID-19 patients.

The currently available data do not provide conclusive insights into the impact of NOAF on the outcomes of critically ill patients in the ICU. Thus, more research is needed to fully understand the prognostic significance of NOAF in relation to ICU and hospital mortality in these patients.

When managing AF in critically ill individuals with both NOAF and COVID-19, it is essential to adhere to existing guidelines while considering the medications administered for COVID-19 treatment. Of course, direct cardioversion should be attempted when there is hemodynamic instability.

## Figures and Tables

**Table 1:** Main findings in NOAF incidence and adverse outcomes of patients in the included studies.

Study	Study Type	MV (%)	*n*	M%	ICU %	NOAF (%)	NOAF Ward/ICU	Age Mean	HF Troponin /Echo	Mortality	HF Difference	Mortality ^1^ No/NOAF
Liu K 2020 [27]	Retrospective	0	137	45	NA	7.3% palpitations		57	NA	11.7%	NA	NA
Angeli F 2020 [10]	Retrospective	0	50	72	0	3	NA	64	NA	NA	NO	NA
Bhatla A 2020 [11]	Retrospective	NA	700	45	11	25	NA	50	Troponin	4%	NA	3/23
Wang Y 2020 [5]	Retrospective	0	319	48	30	20	NA	65	Troponin	NA	NA	NA
Kanthasamy V 2021 [28]	Retrospective	NA	109	83	100	14.6	NA/14.6	59	Troponin + Echo	38%	NO	29/69
Colon C 2020 [13]	Retrospective	NA	115	54	60	16.5 (NOAF + AF)	0/27.5	57.2	Troponin	NA	NA	NA
Kensara R 2023 [29]	Retrospective	74.5	1256	62.3	100	10.7	NA/10.7	61	NA		NA	50.6/63.3
Zakynthinos G 2022 [21]	Prospective	100	79	76	100	24	NA/24	69	Troponin + Echo	45	NO	41.7/47
Ergün B 2021 [30]	Retrospective	NA	248	78	100	14.9	NA/14.9	71	Troponin	NA	NO	67/87
Goyal P 2020 [14]	Retrospective	33	393	60.6	NA	7.1	MV 17.7/ NO MV 1.9	62.2	NA	10.2	NA	NA
Romiti G 2021 [31]	Meta-analysis	NA	187,716	70	NA	8	NA	NA	NA	NA	NA	4-fold increase
Rosenblatt A 2022 [32]	Registry	NA	30,999	51	26	5.4	NA/11.9	61.6	NA	13.6	NA	11.9/45.2
Abdulrahman A 2021 [33]	Retrospective	NA	492	NA	100	6.1	NA/6.1	53.4	Echo	16.3	NO	13.7/56.7
Ip R 2021 [34]	Retrospective	NA	171	57	100	18.7	NA/18.7	66.1	Echo	46.2	NA	40.3/72
Pardo Sanz A 2021 [35]	Retrospective	NA	160	60	4	7.5	NA	65.7	Troponin	18.8	NO	17.6/33.3
Offerhaus J 2022 [36]	Retrospective	NA	5782	64	NA	11	NA	67	NA	16	NA	1.9-fold increase
Li Z 2021 [37]	Meta-analysis	NA	2165 13,075 *	58.7	NA	11	NA		NA	NA	NA	29.3/50.6 *
Russo V 2020 [38]	Retrospective	NA	414	61.1	NA	17.1	NA	66.9	NA	25.8	NA	ND

The table summarizes the main studies included in the review, mainly epidemiological and mortality data in patients presenting NOAF or no NOAF. The heart function is estimated through troponin levels or echocardiographic data (where applicable). AF = atrial flutter; HF = heart function estimated through troponin and/or echocardiogram (if troponin T was above the 99th percentile of the upper reference range); HF = HF difference between patients with NOAF and no NOAF; ICU = patients hospitalized in Intensive Care Unit; M: Male; ^1^ mortality = 30 day or hospital mortality; NOAF and no NOAF; NOAF = new-onset atrial fibrillation; NO = no NOAF; NA = not applicable, ND = no difference; ward = hospitalized patients studied in ward; * = 8 articles with a total of 13,075 participants reported the association between AF and mortality.

## Data Availability

Not applicable.

## References

[1] Phua J., Weng L., Ling L., Egi M., Lim C.M., Divatia J.V., Shrestha B.R., Arabi Y.M., Ng J., Gomersall C.D. (2020). Intensive care management of coronavirus disease 2019 (COVID-19): Challenges and recommendations. Lancet Respir. Med..

[2] Grasselli G., Zangrillo A., Zanella A., Antonelli M., Cabrini L., Castelli A., Cereda D., Coluccello A., Foti G., Fumagalli R. (2020). COVID-19 Lombardy ICU Network. Baseline Characteristics and Outcomes of 1591 Patients Infected with SARS-CoV-2 Admitted to ICUs of the Lombardy Region, Italy. JAMA.

[3] Cummings M.J., Baldwin M.R., Abrams D., Jacobson S.D., Meyer B.J., Balough E.M., Aaron J.G., Claassen J., Rabbani L.E., Hastie J. (2020). Epidemiology, clinical course, and outcomes of critically ill adults with COVID-19 in New York City: A prospective cohort study. Lancet.

[4] Gawałko M., Kapłon-Cieślicka A., Hohl M., Dobrev D., Linz D. (2020). COVID-19 associated atrial fibrillation: Incidence, putative mechanisms and potential clinical implications. Int. J. Cardiol. Heart Vasc..

[5] Wang Y., Shu H., Liu H., Li X., Zhou X., Zou X., Pan S., Xu J., Xu D., Zhao X. (2021). The peak levels of highly sensitive troponin I predicts in-hospital mortality in COVID-19 patients with cardiac injury: A retrospective study. Eur. Heart J. Acute Cardiovasc. Care.

[6] Tsolaki V., Zakynthinos G.E. (2020). Are Patients with COVID-19 Dying of or with Cardiac Injury?. Am. J. Respir. Crit. Care Med..

[7] Siripanthong B., Nazarian S., Muser D., Deo R., Santangeli P., Khanji M.Y., Cooper L.T., Chahal C.A.A. (2020). Recognizing COVID-19-related myocarditis: The possible pathophysiology and proposed guideline for diagnosis and management. Heart Rhythm.

[8] Imazio M., Klingel K., Kindermann I., Brucato A., De Rosa F.G., Adler Y., De Ferrari G.M. (2020). COVID-19 pandemic and troponin: Indirect myocardial injury, myocardial inflammation or myocarditis?. Heart.

[9] Yarmohammadi H., Morrow J.P., Dizon J., Biviano A., Ehlert F., Saluja D., Waase M., Elias P., Poterucha T.J., Berman J. (2021). Frequency of Atrial Arrhythmia in Hospitalized Patients With COVID-19. Am. J. Cardiol..

[10] Angeli F., Spanevello A., De Ponti R., Visca D., Marazzato J., Palmiotto G., Feci D., Reboldi G., Fabbri L.M., Verdecchia P. (2020). Electrocardiographic features of patients with COVID-19 pneumonia. Eur. J. Intern. Med..

[11] Bhatla A., Mayer M.M., Adusumalli S., Hyman M.C., Oh E., Tierney A., Moss J., Chahal A.A., Anesi G., Denduluri S. (2020). COVID-19 and cardiac arrhythmias. Heart Rhythm.

[12] Iacopino S., Placentino F., Colella J., Pesce F., Pardeo A., Filannino P., Artale P., Desiro D., Sorrenti P., Campagna G. (2020). New-Onset Cardiac Arrhythmias during COVID-19 Hospitalization. Circ. Arrhythmia Electrophysiol..

[13] Colon C.M., Barrios J.G., Chiles J.W., McElwee S.K., Russell D.W., Maddox W.R., Kay G.N. (2020). Atrial Arrhythmias in COVID-19 Patients. JACC Clin. Electrophysiol..

[14] Goyal P., Choi J.J., Pinheiro L.C., Schenck E.J., Chen R., Jabri A., Satlin M.J., Campion T.R., Nahid M., Ringel J.B. (2020). Clinical Characteristics of Covid-19 in New York City. N. Engl. J. Med..

[15] Babapoor-Farrokhran S., Rasekhi R.T., Gill D., Babapoor S., Amanullah A. (2020). Arrhythmia in COVID-19. SN Compr. Clin. Med..

[16] Vahey G.M., Marshall K.E., McDonald E., Martin S.W., Tate J.E., Midgley C.M., Killerby M.E., Kawasaki B., Herlihy R.K., Alden N.B. (2021). Symptom Profiles and Progression in Hospitalized and Nonhospitalized Patients with Coronavirus Disease, Colorado, USA, 2020. Emerg. Infect. Dis..

[17] Bosch N.A., Cimini J., Walkey A.J. (2018). Atrial Fibrillation in the ICU. Chest.

[18] Wetterslev M., Haase N., Hassager C., Belley-Cote E.P., McIntyre W.F., An Y., Shen J., Cavalcanti A.B., Zampieri F.G., Guimaraes H.P. (2019). New-onset atrial fibrillation in adult critically ill patients: A scoping review. Intensive Care Med..

[19] Walkey A.J., Benjamin E.J., Lubitz S.A. (2014). New-onset atrial fibrillation during hospitalization. J. Am. Coll. Cardiol..

[20] Arrigo M., Ishihara S., Feliot E., Rudiger A., Deye N., Cariou A., Guidet B., Jaber S., Leone M., Resche-Rigon M. (2018). New-onset atrial fibrillation in critically ill patients and its association with mortality: A report from the FROG-ICU study. Int. J. Cardiol..

[21] Zakynthinos G.E., Tsolaki V., Karavidas N., Vazgiourakis V., Dimeas G., Mantzarlis K., Vavougios G., Makris D. (2022). Secondary bacterial infections are a leading factor triggering New Onset Atrial Fibrillation in intubated ICU COVID-19 ARDS patients. J. Infect. Public Health.

[22] Meierhenrich R., Steinhilber E., Eggermann C., Weiss M., Voglic S., Bögelein D., Gauss A., Georgieff M., Stahl W. (2010). Incidence and prognostic impact of new-onset atrial fibrillation in patients with septic shock: A prospective observational study. Crit. Care.

[23] Salman S., Bajwa A., Gajic O., Afessa B. (2008). Paroxysmal atrial fibrillation in critically ill patients with sepsis. J. Intensive Care Med..

[24] Seguin P., Signouret T., Laviolle B., Branger B., Mallédant Y. (2004). Incidence and risk factors of atrial fibrillation in a surgical intensive care unit. Crit. Care Med..

[25] Manolis A.S., Manolis A.A., Manolis T.A., Apostolopoulos E.J., Papatheou D., Melita H. (2020). COVID-19 infection and cardiac arrhythmias. Trends Cardiovasc. Med..

[26] Nishiga M., Wang D.W., Han Y., Lewis D.B., Wu J.C. (2020). COVID-19 and cardiovascular disease: From basic mechanisms to clinical perspectives. Nat. Rev. Cardiol..

[27] Liu K., Fang Y.Y., Deng Y., Liu W., Wang M.F., Ma J.P., Xiao W., Wang Y.N., Zhong M.H., Li C.H. (2020). Clinical characteristics of novel coronavirus cases in tertiary hospitals in Hubei Province. Chin. Med. J..

[28] Kanthasamy V., Schilling R.J. (2021). Incidence and Prognostic Impact of New-Onset Atrial Fibrillation in Patients with Severe Covid-19: A Retrospective Cohort Study. J. Atr. Fibrillation.

[29] Kensara R., Aljuhani O., Korayem G.B., Alkofide H., Almohareb S.N., Alosaimi Y.S., Altebainawi A.F., Bin Saleh K., Andas N.A., Harbi S.A. (2023). Incidence and Clinical Outcomes of New-Onset Atrial Fibrillation in Critically Ill Patients with COVID-19: A Multicenter Cohort Study—New-Onset Atrial Fibrillation and COVID-19. Clin. Appl. Thromb. Hemost..

[30] Ergün B., Ergan B., Sözmen M.K., Küçük M., Yakar M.N., Cömert B., Gökmen A.N., Yaka E. (2021). New-onset atrial fibrillation in critically ill patients with coronavirus disease 2019 (COVID-19). J. Arrhythmia.

[31] Romiti G.F., Corica B., Lip G.Y.H., Proietti M. (2021). Prevalence and Impact of Atrial Fibrillation in Hospitalized Patients with COVID-19: A Systematic Review and Meta-Analysis. J. Clin. Med..

[32] Gasperetti A., Schiavone M., Tondo C., Mitacchione G., Viecca M., Galli M., Sarzi-Puttini P., Forleo G.B. (2021). QT Interval Monitoring and Drugs Management during COVID-19 Pandemic. Curr. Rev. Clin. Exp. Pharmacol..

[33] Malin J.J., Suárez I., Priesner V., Fätkenheuer G., Rybniker J. (2020). Remdesivir against COVID-19 and Other Viral Diseases. Clin. Microbiol. Rev..

[34] Grein J., Ohmagari N., Shin D., Diaz G., Asperges E., Castagna A., Feldt T., Green G., Green M.L., Lescure F.X. (2020). Compassionate Use of Remdesivir for Patients with Severe Covid-19. N. Engl. J. Med..

[35] Bistrovic P., Lucijanic M. (2021). Remdesivir might induce changes in electrocardiogram beyond bradycardia in patients with coronavirus disease 2019—The pilot study. J. Med. Virol..

[36] Benjamin E.J., Levy D., Vaziri S.M., D’Agostino R.B., Belanger A.J., Wolf P.A. (1994). Independent risk factors for atrial fibrillation in a population-based cohort. The Framingham Heart Study. JAMA.

[37] Roselli C., Rienstra M., Ellinor P.T. (2020). Genetics of Atrial Fibrillation in 2020: GWAS, Genome Sequencing, Polygenic Risk, and Beyond. Circ. Res..

[38] Rosenblatt A.G., Ayers C.R., Rao A., Howell S.J., Hendren N.S., Zadikany R.H., Ebinger J.E., Daniels J.D., Link M.S., de Lemos J.A. (2022). New-Onset Atrial Fibrillation in Patients Hospitalized with COVID-19: Results from the American Heart Association COVID-19 Cardiovascular Registry. Circ. Arrhythmia Electrophysiol..

[39] Gundlund A., Olesen J.B., Butt J.H., Christensen M.A., Gislason G.H., Torp-Pedersen C., Køber L., Kümler T., Fosbøl E.L. (2020). One-year outcomes in atrial fibrillation presenting during infections: A nationwide registry-based study. Eur. Heart J..

[40] Boos C.J. (2020). Infection and atrial fibrillation: Inflammation begets AF. Eur. Heart J..

[41] Walkey A.J., Evans S.R., Winter M.R., Benjamin E.J. (2016). Practice Patterns and Outcomes of Treatments for Atrial Fibrillation during Sepsis: A Propensity-Matched Cohort Study. Chest.

[42] Klein Klouwenberg P.M., Frencken J.F., Kuipers S., Ong D.S., Peelen L.M., van Vught L.A., Schultz M.J., van der Poll T., Bonten M.J., Cremer O.L. (2017). Incidence, Predictors, and Outcomes of New-Onset Atrial Fibrillation in Critically Ill Patients with Sepsis. A Cohort Study. Am. J. Respir. Crit. Care Med..

[43] Artucio H., Pereira M. (1990). Cardiac arrhythmias in critically ill patients: Epidemiologic study. Crit. Care Med..

[44] Tralhão A., Póvoa P. (2020). Cardiovascular Events After Community-Acquired Pneumonia: A Global Perspective with Systematic Review and Meta-Analysis of Observational Studies. J. Clin. Med..

[45] Corica B., Tartaglia F., Oliva A., Raparelli V., Cangemi R., Basili S., Lip G.Y.H., Proietti M., Romiti G.F. (2023). Prevalence of new-onset atrial fibrillation in hospitalized patients with community-acquired pneumonia: A systematic review and meta-analysis. Intern. Emerg. Med..

[46] Søgaard M., Skjøth F., Nielsen P.B., Smit J., Dalager-Pedersen M., Larsen T.B., Lip G.Y.H. (2022). Thromboembolic Risk in Patients with Pneumonia and New-Onset Atrial Fibrillation Not Receiving Anticoagulation Therapy. JAMA Netw. Open.

[47] Kochi A.N., Tagliari A.P., Forleo G.B., Fassini G.M., Tondo C. (2020). Cardiac and arrhythmic complications in patients with COVID-19. J. Cardiovasc. Electrophysiol..

[48] Helms J., Combes A., Aissaoui N. (2022). Cardiac injury in COVID-19. Intensive Care Med..

[49] Zhou F., Yu T., Du R., Fan G., Liu Y., Liu Z., Xiang J., Wang Y., Song B., Gu X. (2020). Clinical course and risk factors for mortality of adult inpatients with COVID-19 in Wuhan, China: A retrospective cohort study. Lancet.

[50] Chen C., Zhou Y., Wang D.W. (2020). SARS-CoV-2: A potential novel etiology of fulminant myocarditis. Herz.

[51] Yang X., Yu Y., Xu J., Shu H., Xia J., Liu H., Wu Y., Zhang L., Yu Z., Fang M. (2020). Clinical course and outcomes of critically ill patients with SARS-CoV-2 pneumonia in Wuhan, China: A single-centered, retrospective, observational study. Lancet Respir. Med..

[52] Tian S., Xiong Y., Liu H., Niu L., Guo J., Liao M., Xiao S.Y. (2020). Pathological study of the 2019 novel coronavirus disease (COVID-19) through postmortem core biopsies. Mod. Pathol..

[53] D’Alto M., Marra A.M., Severino S., Salzano A., Romeo E., De Rosa R., Stagnaro F.M., Pagnano G., Verde R., Murino P. (2020). Right ventricular-arterial uncoupling independently predicts survival in COVID-19 ARDS. Crit. Care.

[54] Bagate F., Masi P., d’Humières T., Al-Assaad L., Chakra L.A., Razazi K., de Prost N., Carteaux G., Derumeaux G., Mekontso Dessap A. (2021). Advanced echocardiographic phenotyping of critically ill patients with coronavirus-19 sepsis: A prospective cohort study. J. Intensive Care.

[55] van den Heuvel F.M.A., Vos J.L., Koop Y., van Dijk A.P.J., Duijnhouwer A.L., de Mast Q., van de Veerdonk F.L., Bosch F., Kok B., Netea M.G. (2020). Cardiac function in relation to myocardial injury in hospitalised patients with COVID-19. Neth. Heart J..

[56] Bieber S., Kraechan A., Hellmuth J.C., Muenchhoff M., Scherer C., Schroeder I., Irlbeck M., Kaeaeb S., Massberg S., Hausleiter J. (2021). Left and right ventricular dysfunction in patients with COVID-19-associated myocardial injury. Infection.

[57] Huang S., Vignon P., Mekontso-Dessap A., Tran S., Prat G., Chew M., Balik M., Sanfilippo F., Banauch G., Clau-Terre F. (2022). Echocardiography findings in COVID-19 patients admitted to intensive care units: A multi-national observational study (the ECHO-COVID study). Intensive Care Med..

[58] Janus S.E., Hajjari J., Karnib M., Tashtish N., Al-Kindi S.G., Hoit B.D. (2020). Prognostic Value of Left Ventricular Global Longitudinal Strain in COVID-19. Am. J. Cardiol..

[59] Karagodin I., Carvalho Singulane C., Woodward G.M., Xie M., Tucay E.S., Tude Rodrigues A.C., Vasquez-Ortiz Z.Y., Alizadehasl A., Monaghan M.J., Ordonez Salazar B.A. (2021). Echocardiographic Correlates of In-Hospital Death in Patients with Acute COVID-19 Infection: The World Alliance Societies of Echocardiography (WASE-COVID) Study. J. Am. Soc. Echocardiogr..

[60] Cheng M.P., Cau A., Lee T.C., Brodie D., Slutsky A., Marshall J., Murthy S., Lee T., Singer J., Demir K.K. (2021). Acute Cardiac Injury in Coronavirus Disease 2019 and Other Viral Infections—A Systematic Review and Meta-Analysis. Crit. Care Med..

[61] Bleakley C., Singh S., Garfield B., Morosin M., Surkova E., Mandalia M.S., Dias B., Androulakis E., Price L.C., McCabe C. (2021). Right ventricular dysfunction in critically ill COVID-19 ARDS. Int. J. Cardiol..

[62] McCall P.J., Willder J.M., Stanley B.L., Messow C.M., Allan J., Gemmell L., Puxty A., Strachan D., Berry C., Shelley B.G. (2022). Right ventricular dysfunction in patients with COVID-19 pneumonitis whose lungs are mechanically ventilated: A multicentre prospective cohort study. Anaesthesia.

[63] Corica B., Marra A.M., Basili S., Cangemi R., Cittadini A., Proietti M., Romiti G.F. (2021). Prevalence of right ventricular dysfunction and impact on all-cause death in hospitalized patients with COVID-19: A systematic review and meta-analysis. Sci. Rep..

[64] Evrard B., Goudelin M., Giraudeau B., François B., Vignon P. (2022). Right ventricular failure is strongly associated with mortality in patients with moderate-to-severe COVID-19-related ARDS and appears related to respiratory worsening. Intensive Care Med..

[65] Karagodin I., Singulane C.C., Descamps T., Woodward G.M., Xie M., Tucay E.S., Sarwar R., Vasquez-Ortiz Z.Y., Alizadehasl A., Monaghan M.J. (2022). Ventricular Changes in Patients with Acute COVID-19 Infection: Follow-up of the World Alliance Societies of Echocardiography (WASE-COVID) Study. J. Am. Soc. Echocardiogr..

[66] Bursi F., Santangelo G., Sansalone D., Valli F., Vella A.M., Toriello F., Barbieri A., Carugo S. (2020). Prognostic utility of quantitative offline 2D-echocardiography in hospitalized patients with COVID-19 disease. Echocardiography.

[67] Li Y., Li H., Zhu S., Xie Y., Wang B., He L., Zhang D., Zhang Y., Yuan H., Wu C. (2020). Prognostic Value of Right Ventricular Longitudinal Strain in Patients With COVID-19. JACC Cardiovasc. Imaging.

[68] Slobod D., Assanangkornchai N., Alhazza M., Mettasittigorn P., Magder S. (2022). Right Ventricular Loading by Lung Inflation during Controlled Mechanical Ventilation. Am. J. Respir. Crit. Care Med..

[69] Schmitt J.M., Vieillard-Baron A., Augarde R., Prin S., Page B., Jardin F. (2001). Positive end-expiratory pressure titration in acute respiratory distress syndrome patients: Impact on right ventricular outflow impedance evaluated by pulmonary artery Doppler flow velocity measurements. Crit. Care Med..

[70] Protti A., Santini A., Pennati F., Chiurazzi C., Cressoni M., Ferrari M., Iapichino G.E., Carenzo L., Lanza E., Picardo G. (2022). Lung Response to a Higher Positive End-Expiratory Pressure in Mechanically Ventilated Patients with COVID-19. Chest.

[71] Tsolaki V., Zakynthinos G.E., Mantzarlis K., Vazgiourakis V., Makris D. (2021). Pathophysiology of COVID-19-associated acute respiratory distress syndrome. Lancet Respir. Med..

[72] Tsolaki V., Zakynthinos G.E., Papanikolaou J., Karavidas N., Vazgiourakis V., Papadonta M.E., Zygoulis P., Pantazopoulos I., Makris D., Zakynthinos E. (2023). Positive End-Expiratory Pressure Deescalation in COVID-19-induced Acute Respiratory Distress Syndrome Unloads the Right Ventricle, Improving Hemodynamics and Oxygenation. Am. J. Respir. Crit. Care Med..

[73] Kawakami R., Sakamoto A., Kawai K., Gianatti A., Pellegrini D., Nasr A., Kutys B., Guo L., Cornelissen A., Mori M. (2021). Pathological Evidence for SARS-CoV-2 as a Cause of Myocarditis: JACC Review Topic of the Week. J. Am. Coll. Cardiol..

[74] Kim J.S., Kim M., Kim Y.J., Ryoo S.M., Sohn C.H., Ahn S., Kim W.Y. (2019). Troponin Testing for Assessing Sepsis-Induced Myocardial Dysfunction in Patients with Septic Shock. J. Clin. Med..

[75] Wu A.H. (2001). Increased troponin in patients with sepsis and septic shock: Myocardial necrosis or reversible myocardial depression?. Intensive Care Med..

[76] Jeong H.S., Lee T.H., Bang C.H., Kim J.H., Hong S.J. (2018). Risk factors and outcomes of sepsis-induced myocardial dysfunction and stress-induced cardiomyopathy in sepsis or septic shock: A comparative retrospective study. Medicine.

[77] Vieillard-Baron A., Caille V., Charron C., Belliard G., Page B., Jardin F. (2008). Actual incidence of global left ventricular hypokinesia in adult septic shock. Crit. Care Med..

[78] Spathoulas K., Tsolaki V., Zakynthinos G.E., Karelas D., Makris D., Zakynthinos E., Papanikolaou J. (2022). The Role of Left Ventricular Ejection Fraction and Left Ventricular Outflow Tract Velocity-Time Integral in Assessing Cardiovascular Impairment in Septic Shock. J. Pers. Med..

[79] Seguin P., Laviolle B., Maurice A., Leclercq C., Mallédant Y. (2006). Atrial fibrillation in trauma patients requiring intensive care. Intensive Care Med..

[80] Walkey A.J., Wiener R.S., Ghobrial J.M., Curtis L.H., Benjamin E.J. (2011). Incident stroke and mortality associated with new-onset atrial fibrillation in patients hospitalized with severe sepsis. JAMA.

[81] Walkey A.J., Greiner M.A., Heckbert S.R., Jensen P.N., Piccini J.P., Sinner M.F., Curtis L.H., Benjamin E.J. (2013). Atrial fibrillation among Medicare beneficiaries hospitalized with sepsis: Incidence and risk factors. Am. Heart J..

[82] Vieillard-Baron A., Boyd J. (2018). Non-antiarrhythmic interventions in new onset and paroxysmal sepsis-related atrial fibrillation. Intensive Care Med..

[83] Schotten U., Verheule S., Kirchhof P., Goette A. (2011). Pathophysiological mechanisms of atrial fibrillation: A translational appraisal. Physiol. Rev..

[84] Seemann A., Boissier F., Razazi K., Carteaux G., de Prost N., Brun-Buisson C., Mekontso Dessap A. (2015). New-onset supraventricular arrhythmia during septic shock: Prevalence, risk factors and prognosis. Ann. Intensive Care.

[85] Yao C., Veleva T., Scott L., Cao S., Li L., Chen G., Jeyabal P., Pan X., Alsina K.M., Abu-Taha I. (2018). Enhanced Cardiomyocyte NLRP3 Inflammasome Signaling Promotes Atrial Fibrillation. Circulation.

[86] Sirish P., Li N., Timofeyev V., Zhang X.D., Wang L., Yang J., Lee K.S., Bettaieb A., Ma S.M., Lee J.H. (2016). Molecular Mechanisms and New Treatment Paradigm for Atrial Fibrillation. Circ. Arrhythmia Electrophysiol..

[87] Donniacuo M., De Angelis A., Rafaniello C., Cianflone E., Paolisso P., Torella D., Sibilio G., Paolisso G., Castaldo G., Urbanek K. (2023). COVID-19 and atrial fibrillation: Intercepting lines. Front. Cardiovasc. Med..

[88] Libby P., Lüscher T. (2020). COVID-19 is, in the end, an endothelial disease. Eur. Heart J..

[89] Manjili R.H., Zarei M., Habibi M., Manjili M.H. (2020). COVID-19 as an Acute Inflammatory Disease. J. Immunol..

[90] Salama C., Han J., Yau L., Reiss W.G., Kramer B., Neidhart J.D., Criner G.J., Kaplan-Lewis E., Baden R., Pandit L. (2021). Tocilizumab in Patients Hospitalized with Covid-19 Pneumonia. N. Engl. J. Med..

[91] Huang Q., Wu X., Zheng X., Luo S., Xu S., Weng J. (2020). Targeting inflammation and cytokine storm in COVID-19. Pharmacol. Res..

[92] Gerotziafas G.T., Catalano M., Colgan M.P., Pecsvarady Z., Wautrecht J.C., Fazeli B., Olinic D.M., Farkas K., Elalamy I., Falanga A. (2020). Guidance for the Management of Patients with Vascular Disease or Cardiovascular Risk Factors and COVID-19: Position Paper from VAS-European Independent Foundation in Angiology/Vascular Medicine. Thromb. Haemost..

[93] Gencer S., Lacy M., Atzler D., van der Vorst E.P.C., Döring Y., Weber C. (2020). Immunoinflammatory, Thrombohaemostatic, and Cardiovascular Mechanisms in COVID-19. Thromb. Haemost..

[94] Bikdeli B., Madhavan M.V., Gupta A., Jimenez D., Burton J.R., Der Nigoghossian C., Chuich T., Nouri S.N., Dreyfus I., Driggin E. (2020). Pharmacological Agents Targeting Thromboinflammation in COVID-19: Review and Implications for Future Research. Thromb. Haemost..

[95] Zylla M.M., Merle U., Vey J.A., Korosoglou G., Hofmann E., Müller M., Herth F., Schmidt W., Blessing E., Göggelmann C. (2021). Predictors and Prognostic Implications of Cardiac Arrhythmias in Patients Hospitalized for COVID-19. J. Clin. Med..

[96] Huang C., Wang Y., Li X., Ren L., Zhao J., Hu Y., Zhang L., Fan G., Xu J., Gu X. (2020). Clinical features of patients infected with 2019 novel coronavirus in Wuhan, China. Lancet.

[97] Wong C.K., Lam C.W., Wu A.K., Ip W.K., Lee N.L., Chan I.H., Lit L.C., Hui D.S., Chan M.H., Chung S.S. (2004). Plasma inflammatory cytokines and chemokines in severe acute respiratory syndrome. Clin. Exp. Immunol..

[98] Zeng Z., Yu H., Chen H., Qi W., Chen L., Chen G., Yan W., Chen T., Ning Q., Han M. (2020). Longitudinal changes of inflammatory parameters and their correlation with disease severity and outcomes in patients with COVID-19 from Wuhan, China. Crit. Care.

[99] Buetti N., Ruckly S., de Montmollin E., Reignier J., Terzi N., Cohen Y., Siami S., Dupuis C., Timsit J.F. (2021). COVID-19 increased the risk of ICU-acquired bloodstream infections: A case-cohort study from the multicentric OUTCOMEREA network. Intensive Care Med..

[100] Rouzé A., Martin-Loeches I., Povoa P., Makris D., Artigas A., Bouchereau M., Lambiotte F., Metzelard M., Cuchet P., Boulle Geronimi C. (2021). Relationship between SARS-CoV-2 infection and the incidence of ventilator-associated lower respiratory tract infections: A European multicenter cohort study. Intensive Care Med..

[101] Goudis C.A., Kallergis E.M., Vardas P.E. (2012). Extracellular matrix alterations in the atria: Insights into the mechanisms and perpetuation of atrial fibrillation. Europace.

[102] Rodrigues R., Costa de Oliveira S. (2021). The Impact of *Angiotensin-Converting Enzyme 2* (*ACE2*) Expression Levels in Patients with Comorbidities on COVID-19 Severity: A Comprehensive Review. Microorganisms.

[103] Behl T., Kaur I., Aleya L., Sehgal A., Singh S., Sharma N., Bhatia S., Al-Harrasi A., Bungau S. (2022). CD147-spike protein interaction in COVID-19: Get the ball rolling with a novel receptor and therapeutic target. Sci. Total Environ..

[104] Wang K., Chen W., Zhang Z., Deng Y., Lian J.Q., Du P., Wei D., Zhang Y., Sun X.X., Gong L. (2020). CD147-spike protein is a novel route for SARS-CoV-2 infection to host cells. Signal Transduct. Target. Ther..

[105] Pituch-Noworolska A.M. (2022). NK cells in SARS-CoV-2 infection. Cent. Eur. J. Immunol..

[106] Li X., Guo X., Chang Y., Zhang N., Sun Y. (2022). Analysis of alterations of serum inflammatory cytokines and fibrosis makers in patients with essential hypertension and left ventricular hypertrophy and the risk factors. Am. J. Transl. Res..

[107] Ueland T., Holter J.C., Holten A.R., Müller K.E., Lind A., Bekken G.K., Dudman S., Aukrust P., Dyrhol-Riise A.M., Heggelund L. (2020). Distinct and early increase in circulating MMP-9 in COVID-19 patients with respiratory failure. J. Infect..

[108] Peng X., Wang Y., Xi X., Jia Y., Tian J., Yu B., Tian J. (2021). Promising Therapy for Heart Failure in Patients with Severe COVID-19: Calming the Cytokine Storm. Cardiovasc. Drugs Ther..

[109] Clementy N., Piver E., Bisson A., Andre C., Bernard A., Pierre B., Fauchier L., Babuty D. (2018). Galectin-3 in Atrial Fibrillation: Mechanisms and Therapeutic Implications. Int. J. Mol. Sci..

[110] Cannavo A., Liccardo D., Gelzo M., Amato F., Gentile I., Pinchera B., Femminella G.D., Parrella R., DE Rosa A., Gambino G. (2022). Serum galectin-3 and aldosterone: Potential biomarkers of cardiac complications in patients with COVID-19. Minerva Endocrinol..

[111] Cervantes-Alvarez E., la Rosa N.L., la Mora M.S., Valdez-Sandoval P., Palacios-Jimenez M., Rodriguez-Alvarez F., Vera-Maldonado B.I., Aguirre-Aguilar E., Escobar-Valderrama J.M., Alanis-Mendizabal J. (2022). Galectin-3 as a potential prognostic biomarker of severe COVID-19 in SARS-CoV-2 infected patients. Sci. Rep..

[112] Che Mohd Nassir C.M.N., Zolkefley M.K.I., Ramli M.D., Norman H.H., Abdul Hamid H., Mustapha M. (2022). Neuroinflammation and COVID-19 Ischemic Stroke Recovery-Evolving Evidence for the Mediating Roles of the ACE2/Angiotensin-(1-7)/Mas Receptor Axis and NLRP3 Inflammasome. Int. J. Mol. Sci..

[113] Young L.J., Antwi-Boasiako S., Ferrall J., Wold L.E., Mohler P.J., El Refaey M. (2022). Genetic and non-genetic risk factors associated with atrial fibrillation. Life Sci..

[114] Saljic A., Heijman J., Dobrev D. (2022). Emerging Antiarrhythmic Drugs for Atrial Fibrillation. Int. J. Mol. Sci..

[115] Farahani M., Niknam Z., Mohammadi Amirabad L., Amiri-Dashatan N., Koushki M., Nemati M., Danesh Pouya F., Rezaei-Tavirani M., Rasmi Y., Tayebi L. (2022). Molecular pathways involved in COVID-19 and potential pathway-based therapeutic targets. Biomed. Pharmacother..

[116] Aleebrahim-Dehkordi E., Molavi B., Mokhtari M., Deravi N., Fathi M., Fazel T., Mohebalizadeh M., Koochaki P., Shobeiri P., Hasanpour-Dehkordi A. (2022). T helper type (Th1/Th2) responses to SARS-CoV-2 and influenza A (H1N1) virus: From cytokines produced to immune responses. Transpl. Immunol..

[117] Mazzoni A., Salvati L., Maggi L., Annunziato F., Cosmi L. (2021). Hallmarks of immune response in COVID-19: Exploring dysregulation and exhaustion. Semin. Immunol..

[118] Li S., Wang J., Yan Y., Zhang Z., Gong W., Nie S. (2022). Clinical Characterization and Possible Pathological Mechanism of Acute Myocardial Injury in COVID-19. Front. Cardiovasc. Med..

[119] Fischer L., Barop H., Ludin S.M., Schaible H.G. (2022). Regulation of acute reflectory hyperinflammation in viral and other diseases by means of stellate ganglion block. A conceptual view with a focus on Covid-19. Auton. Neurosci. Basic Clin..

[120] Bers D.M. (2002). Cardiac excitation-contraction coupling. Nature.

[121] Abdulrahman A., Hussain T., Nawaz S., AlShaikh S., Almadani A., Bardooli F. (2021). Is Atrial Fibrillation a Risk Factor for Worse Outcomes in Severe COVID-19 Patients: A Single Center Retrospective Cohort. J. Saudi Heart Assoc..

[122] Ip R.J., Ali A., Baloch Z.Q., Al-Abcha A., Jacob C., Arnautovic J., Boumegouas M., Do S., Meka K., Wilcox M. (2021). Atrial Fibrillation as a Predictor of Mortality in High Risk COVID-19 Patients: A Multicentre Study of 171 Patients. Heart Lung Circ..

[123] Pardo Sanz A., Salido Tahoces L., Ortega Pérez R., González Ferrer E., Sánchez Recalde Á., Zamorano Gómez J.L. (2021). New-onset atrial fibrillation during COVID-19 infection predicts poor prognosis. Cardiol. J..

[124] Offerhaus J.A., Joosten L.P.T., van Smeden M., Linschoten M., Bleijendaal H., Tieleman R., Wilde A.A.M., Rutten F.H., Geersing G.J., Remme C.A. (2022). Sex- and age specific association of new-onset atrial fibrillation with in-hospital mortality in hospitalised COVID-19 patients. Int. J. Cardiol. Heart Vasc..

[125] Li Z., Shao W., Zhang J., Ma J., Huang S., Yu P., Zhu W., Liu X. (2021). Prevalence of Atrial Fibrillation and Associated Mortality Among Hospitalized Patients with COVID-19: A Systematic Review and Meta-Analysis. Front. Cardiovasc. Med..

[126] Russo V., Di Maio M., Mottola F.F., Pagnano G., Attena E., Verde N., Di Micco P., Silverio A., Scudiero F., Nunziata L. (2020). Clinical characteristics and prognosis of hospitalized COVID-19 patients with incident sustained tachyarrhythmias: A multicenter observational study. Eur. J. Clin. Investig..

[127] Liu W.C., Lin W.Y., Lin C.S., Huang H.B., Lin T.C., Cheng S.M., Yang S.P., Lin J.C., Lin W.S. (2016). Prognostic impact of restored sinus rhythm in patients with sepsis and new-onset atrial fibrillation. Crit. Care.

[128] Wang T.J., Larson M.G., Levy D., Vasan R.S., Leip E.P., Wolf P.A., D’Agostino R.B., Murabito J.M., Kannel W.B., Benjamin E.J. (2003). Temporal relations of atrial fibrillation and congestive heart failure and their joint influence on mortality: The Framingham Heart Study. Circulation.

[129] Annane D., Sébille V., Duboc D., Le Heuzey J.Y., Sadoul N., Bouvier E., Bellissant E. (2008). Incidence and prognosis of sustained arrhythmias in critically ill patients. Am. J. Respir. Crit. Care Med..

[130] Carrera P., Thongprayoon C., Cheungpasitporn W., Iyer V.N., Moua T. (2016). Epidemiology and outcome of new-onset atrial fibrillation in the medical intensive care unit. J. Crit. Care.

[131] Shaver C.M., Chen W., Janz D.R., May A.K., Darbar D., Bernard G.R., Bastarache J.A., Ware L.B. (2015). Atrial Fibrillation Is an Independent Predictor of Mortality in Critically Ill Patients. Crit. Care Med..

[132] Manolis A.S. (2015). Rhythm or Rate Control Management of Atrial Fibrillation: An Overrated Dilemma. Hell. J. Cardiol..

[133] Zimetbaum P. (2012). Antiarrhythmic drug therapy for atrial fibrillation. Circulation.

[134] Kirchhof P., Benussi S., Kotecha D., Ahlsson A., Atar D., Casadei B., Castella M., Diener H.C., Heidbuchel H., Hendriks J. (2016). 2016 ESC Guidelines for the management of atrial fibrillation developed in collaboration with EACTS. Europace.

[135] Manolis A.S., Manolis T.A. (2020). Cardiovascular complications of the coronavirus (COVID-19) infection. Rhythmos.

[136] Lydtin H. (1977). Side effects and contraindications of beta-receptor blocking agents. Klin. Wochenschr..

[137] Van Gelder I.C., Rienstra M., Crijns H.J., Olshansky B. (2016). Rate control in atrial fibrillation. Lancet.

[138] Wetterslev M., Hylander Møller M., Granholm A., Hassager C., Haase N., Lange T., Myatra S.N., Hästbacka J., Arabi Y.M., Shen J. (2023). Atrial Fibrillation (AFIB) in the ICU: Incidence, Risk Factors, and Outcomes: The International AFIB-ICU Cohort Study. Crit. Care Med..

[139] Goette A., Brandner S. (2022). Atrial fibrillation on the intensive care unit: The special prognostic importance of the first manifestation. Herzschrittmachertherapie Elektrophysiologie.

[140] Wetterslev M., Møller M.H., Granholm A., Hassager C., Haase N., Aslam T.N., Shen J., Young P.J., Aneman A., Hästbacka J. (2022). Management of acute atrial fibrillation in the intensive care unit: An international survey. Acta Anaesthesiol. Scand..

[141] Bedford J., Drikite L., Corbett M., Doidge J., Ferrando-Vivas P., Johnson A., Rajappan K., Mouncey P., Harrison D., Young D. (2021). Pharmacological and non-pharmacological treatments and outcomes for new-onset atrial fibrillation in ICU patients: The CAFE scoping review and database analyses. Health Technol. Assess..

[142] Kaufman E.S., Zimmermann P.A., Wang T., Dennish G.W., Barrell P.D., Chandler M.L., Greene H.L., Atrial Fibrillation Follow-up Investigation of Rhythm Management Investigators (2004). Risk of proarrhythmic events in the Atrial Fibrillation Follow-up Investigation of Rhythm Management (AFFIRM) study: A multivariate analysis. J. Am. Coll. Cardiol..

[143] Manolis A.S., Tordjman T., Mack K.D., Estes N.A. (1987). Atypical pulmonary and neurologic complications of amiodarone in the same patient. Report of a case and review of the literature. Arch. Intern. Med..

[144] Manolis A.S. (2012). Did PALLAS deliver the final blow to dronedarone?. Rhythmos.

[145] Chiladakis J.A., Kalogeropoulos A., Patsouras N., Manolis A.S. (2004). Ibutilide added to propafenone for the conversion of atrial fibrillation and atrial flutter. J. Am. Coll. Cardiol..

[146] Manolis A.S., Bethanis S., Metaxa S., Polytarchou K., Sakellaris N., Pyrros I. (2019). Use of intravenous vernakalant for atrial fibrillation conversion in the regular ward under only bedside monitoring. Hell. J. Cardiol..

[147] Pantazopoulos I., Mavrovounis G., Dimeas G., Zikos N., Pitsikou M., Rousogianni E., Mermiri M., Michou A., Spanos M., Maniotis C. (2022). Remdesivir-induced Bradycardia is not Associated with Worse Outcome in Patients with COVID-19: A Retrospective Analysis. Am. J. Cardiovasc. Drugs.

[148] Berman J.P., Abrams M.P., Kushnir A., Rubin G.A., Ehlert F., Biviano A., Morrow J.P., Dizon J., Wan E.Y., Yarmohammadi H. (2020). Cardiac electrophysiology consultative experience at the epicenter of the COVID-19 pandemic in the United States. Indian Pacing Electrophysiol. J..

[149] January C.T., Wann L.S., Alpert J.S., Calkins H., Cigarroa J.E., Cleveland J.C., Conti J.B., Ellinor P.T., Ezekowitz M.D., Field M.E. (2014). 2014 AHA/ACC/HRS guideline for the management of patients with atrial fibrillation: A report of the American College of Cardiology/American Heart Association Task Force on practice guidelines and the Heart Rhythm Society. Circulation.

